# Friends or foes? A compatibility assessment of bioeconomy-related Sustainable Development Goals for European policy coherence

**DOI:** 10.1016/j.jclepro.2019.119832

**Published:** 2020-05-01

**Authors:** Tévécia Ronzon, Ana I. Sanjuán

**Affiliations:** a European Commission, Joint Research Centre (JRC), Seville, Spain; b Agricultural Economics and Rural Policy Group, Wageningen University, the Netherlands; c Agrifood Research and Technology Center of Aragon (Agrifood Institute of Aragon, IA2-CITA-Universidad de Zaragoza), Zaragoza, Spain

**Keywords:** Bioeconomy, SDG, Policy coherence, Correlation, Biomass

## Abstract

In October 2018, the European Union (EU) launched an updated bioeconomy strategy with the aim of encouraging the substitution of fossil carbon with biomass feedstock in the industry and in energy production while preserving ecosystem services. The objective of the paper is to analyse the links between the EU bioeconomy strategy and the Sustainable Development Goals (SDGs), and to assess what could be the main points of synergies and tensions between bioeconomy-related SDG targets. By semantically mapping the action plan of the 2018 EU bioeconomy strategy with the SDG targets, the paper finds that the bioeconomy strategy is aligned with 53 targets distributed in 12 of the 17 SDGs. Ex-post correlation analysis on bioeconomy-related SDGs indicators for 28 EU Member States (1990–2018) shows a predominance of synergies over trade-offs. More intense synergetic past developments (positive correlations) are found among clean energies (SDG 7), recycling (SDG 11), ecosystem preservation (SDG 15) and most of all other bioeconomy-related SDGs. Negative correlations are observed between agro-biodiversity (SDG 2), domestic material consumption of biomass (SDG 8 and 12), agriculture and industrial developments (SDG 2 and SDG 9) and a wide array of bioeconomy-related SDG indicators. The hotspots of strong correlations identified might be useful in further enrichment of ex-ante simulation models. From a policy coherence perspective, a wide range of policy instruments are already in place in the EU to foster synergies and may bring co-benefits. Policies oriented at preventing trade-offs are already in place but they have not overcome the antagonisms observed in this study yet. Change in practices, technical and technological innovations and the application of circular and ‘cascading principles’ are the most common fields of action.

## Highlights

• The European bioeconomy strategy is aligned with 12 Sustainable Development Goals.
• Synergies predominate over trade-offs in bioeconomy domains (period 1990–2018).
• Main synergies are found with goals 7 (energy), 11 (recycling) and 15 (land).
• Many co-benefits are thus expected from policy measures in these areas.
• Trade-offs with Sustainable Development Goals 2, 8, 12 and 9 require policy attention.

## 1 Introduction

International leaders have committed in 2015 to achieving a set of 17 Sustainable Development Goals (SDGs) by 2030 with the aim of improving the capacity of human beings to live well on a healthy planet. The 17 SDGs, further defined through 169 SDG targets, form a system of interacting components that reflects the highly complex and multi-dimensional nature of sustainable development (United Nations, 2015; Pradhan, 2019). In the European Union (EU), the bioeconomy concept has been put forward since 2012 as an option matching with the pursuit of many aspects of sustainable development, and therefore with a number of SDG targets as defined later in 2015. Generally speaking, the bioeconomy refers to the substitution of fossil carbon with biomass feedstock in the industry and in energy production while preserving ecosystem services.^1^

The first EU bioeconomy strategy, launched in 2012, has nevertheless been criticised for its unbalanced contribution to sustainable development by those who found the strategy oriented too much towards market (Bugge et al., 2016; Scordato et al., 2017) and technology (De Besi and McCormick, 2015; Hausknost et al., 2017), and neglecting the multi-functionality of biomass production (Brunori, 2013; Ramcilovic-Suominen and Pülzl, 2018). Questions were also raised regarding the sustainability of biomass production systems and the capacity of the EU to dispose of enough biomass to meet growing uses (Kovacs, 2015; Lewandowski, 2015; Müller et al., 2015; Giljum et al., 2016; Meyer, 2017; Birner, 2018; Heimann, 2019) as well as the supposed environmental benefits of bio-based products (Bennich and Belyazid, 2017; Priefer et al., 2017; OECD, 2018; Alpizar et al., 2019).

Thus, in 2018, the revision of the EU bioeconomy strategy put emphasis on balancing the three pillars of sustainable development. This effort is evidenced in the title of the revised strategy itself: “A sustainable bioeconomy for Europe: strengthening the connection between economy, society and the environment” (European Commission, 2018b). The revision also integrated a number of former criticisms by attributing equal importance (i) to the scaling-up of bio-based sectors and (ii) to the understanding of the ecological boundaries of the bioeconomy, as well as (iii) to the development of the bioeconomy at the local level as a way to better distribute the benefits of bioeconomy development. Moreover, ecosystem services were explicitly integrated into the new definition of the bioeconomy.

These adjustments have unquestionably reinforced the coherence between the bioeconomy strategy and the EU engagement towards the SDGs. But the examination of the inter-linkages taking place between the multiple objectives of the two initiatives remains a necessary step on the road towards more policy coherence and effectiveness (Lu et al., 2015; Boas et al., 2016; Karnib, 2017; Allen et al., 2018). Indeed, identifying and understanding inter-linkages would help to leverage positive interactions and foster the necessary transformative changes to avoid that progress towards one SDG target rolls back another one (Pradhan et al., 2017; Muscaglia and Miola, 2018; Liu et al., 2019).

Accordingly, the objective of this study is to provide a quantitative assessment of synergies and trade-offs as observed among the SDG indicators that relate to the domains of actions of the new EU bioeconomy strategy. With this assessment, the aim of the paper is two-fold: (i) to provide a stepping stone for the incorporation of non-market SDG indicators^2^ into simulation modelling frameworks through the inter-linkages observed with market-based SDG indicators; and (ii) to provide data-based evidence in support for policy coherence into future EU policy designs. The paper consecutively investigates two research questions: (1) What SDGs and SDG targets does the EU bioeconomy strategy contribute to? and (2) What are the points of synergies and tensions between bioeconomy-related SDG targets?

As described in section 2, to answer the first research question, the actions foreseen in the action plan of the current EU bioeconomy strategy are mapped against the SDG targets that share the same objective (semantic mapping). Regarding the second research question, a statistical *ex-post* analysis of the interactions between bioeconomy-related SDG indicators (i.e. the indicators corresponding to the SDG targets mapped in the first research question) is performed, following closely Pradhan et al. (2017)’s approach that relies on bivariate correlations. The analysis is based on official UN and Eurostat data on SDG indicators, corresponding to 28 EU Member States (MS) over the period 1990–2018 (upon data availability for each SDG indicator). Section 3 presents the results on the mapping of the action plan and SDG targets as well as on the resulting synergies and trade-offs found. Section 4 discusses the quantitative results in the context of the literature and previous knowledge as well as of current and past EU policy actions. The discussion is driven by the two following questions: (1) are the correlations found in this study supported by empirical observations or studies? And (2) if yes, has this knowledge played a role in policy making? Section 5 concludes on the two research questions investigated.

## 2 Data and methods

### 2.1 Data

This study builds on a dataset of bioeconomy-related indicators compiled from the long list of official UN and Eurostat SDG databases (Eurostat, 2019; United Nations Statistics Division, 2019). The “bioeconomy-related” nature of the selected indicators is established after a semantic mapping of the EU bioeconomy action plan with the SDG targets it contributes to. In other words, a SDG target is considered “bioeconomy-related” when a meaning-based equivalence or similarity could be identified between this target and one or several actions of the EU bioeconomy action plan.

The 2018 EU bioeconomy strategy is composed of 23 sub-actions while the 17 SDGs are defined through 169 targets which are in turn measured through multiple indicators. As a sub-action can match more than one SDG target, the complexity of the mapping exercise was reduced by grouping all measures and objectives announced in the action plan into 13 “matching fields” (see supplementary materials (SM): column 2 of Table SM1) that are subsequently mapped to the SDG targets.^3^ The direct correspondence between sub-actions and SDG targets is then cross-checked and presented on Table SM2. The matching exercise does not consider the indirect consequences of a given action. For example, action 2.2 on “pilot actions for a local bioeconomy development” is related to the matching field “Local development” and subsequently to targets belonging to SDGs 2, 8, 9 and 14. One might argue that by contributing to local development, action 2.2 will also have an impact on local levels of poverty (SDG 1) and inequality (SDG 10). As the action does not mention these specific aims, it has not been mapped to SDG 1 and SDG 10.

Identified “bioeconomy-related” SDG targets are measured with 268 UN and Eurostat indicators (i.e. variables) to which a series of filters is applied in order to: (i) keep only indicators with at least three observations per EU Member State between 1990 and 2018^4^; (ii) remove non-relevant indicators from the bioeconomy action plan perspective,^5^ and (iii) avoid the multiple representation of the same indicator expressed in different dimensions or measurement units.^6^ As a result, the final dataset comprises 54 bioeconomy-related SDG indicators, 26 of which come from the UN and the rest from Eurostat.

### 2.2 Statistical tests

Diverse quantitative and qualitative methodologies for the analysis of SDG inter-linkages are described in the scientific literature. They highlight the context specificity of inter-linkages as different directions and strengths are found for the same interaction over time and countries (Nilsson et al., 2016; Pradhan et al., 2017; van Leeuwen, 2017; Zhou and Moinuddin, 2017; Weitz et al., 2018).

This study presents a data-driven approach consisting of bivariate correlations between unique pairs of SDG indicator time series for each EU Member State. Such approach allows the capture of country specificities and the coefficient of correlation informs on the strength of the interaction. In particular, the Spearman rank correlation (ρ) is used as recommended by previous studies on SDGs interactions, such as Pradhan et al. (2017) at the global level; Zhou and Moinuddin (2017) on nine selected Asian countries; and Muscaglia and Miola (2018) on the EU. In comparison to other correlation statistics (i.e. Pearson), the Spearman correlation does not impose the normality assumption, is suitable for non-linear relations and is little sensitive to outliers.

The analysis is carried out sequentially. First, following closely the methodological approach of Pradhan et al. (2017), indicators are checked for consistency in their interpretation previous to correlation analysis. That is, a positive sign is assigned to those indicators whose increase contributes to narrowing the distance to the official SDG target, and a negative sign is assigned to the ones whose increase widens the distance to the target (Table SM3). As a result, positive (and statistically significant) correlations show a synergetic effect for the achievement of SDG targets and negative correlations highlight trade-offs.

Second, pairwise correlation tests are carried out between all bioeconomy-related SDG indicators time series identified in section 2.1. (54), for each of the 28 EU MS. As a result, 28 symmetric matrices of size 54 × 54 are obtained.

Third, only statistically significant correlations at the 5-percent significance level (p-value < 0.05) calculated on more than three years were considered for further analysis. Likewise, using 0.6 as a cutting point (as in Pradhan et al., 2017), pairwise correlations were classified as indicating either synergies (ρ > 0.6), trade-offs (ρ < −0.6) or non-classified (−0.6<ρ < 0.6) relations.

Fourth, the correlation results are aggregated at the EU level by calculating the percentage of synergies, trade-offs and non-classified relations found over the 28 MSs for each pair of indicators and for each SDG pair (intra-SDG and inter-SDG):

(i) SDG results summarize bioeconomy-related SDG intra- and inter-linkages. Among indicators of the same SDG pair (*s,t*), more weight is given to those pairs of indicators (*i,j*) with more significant correlations across the 28 MS. Weights (w_i,j_) are calculated as $w_{i,j}=\frac{N_{i,j}}{\sum_{i}\sum_{j}N_{i,j}}$ , where *N*_*i,j*_ is the count of significant pairwise correlations between bio-economic indicators *i* and *j* (*i≠j*) across countries. N_i,j_ can range up to 28 (i.e. the number of EU countries).
Therefore, the proportion of synergies $SYN_{s}^{t}$ between SDG *s* and SDG *t (s = t* for intra-SDG calculations; *s≠t* for inter-SDG calculations*)* is:

$$SYN_{s}^{t}=\frac{\sum_{i}\sum_{j}S_{i,j}\times w_{i,j}}{\sum_{i}\sum_{j}N_{i,j}\times w_{i,j}}$$

where S_i,j_ is the count of significant synergies between indicators *i* and *j* across countries.

Similarly, by replacing S_i,j_ with the count of trade-offs and non-classified cases, the respective proportion are obtained.
(ii) Indicator pair results are used to identify specific hotspots of trade-offs. A hotspot of trade-off refers to a pattern of negative correlations found in a high proportion across the 28 MSs (i.e. more than 50% of pairwise significant correlations) between a given indicator (or two) and a series of other SDG indicators.
This particular analysis on the indicator pair level might prove useful in the identification of pairs of non-market and market indicators with stronger links that can, later on, contribute to the enrichment of ex-ante simulation models with more non-market outcome variables.

Results at both levels of analysis (SDG pair level and indicator pair level) will contribute to the second aim of the paper which is to provide data-based evidence in support to policy coherence into future EU policy designs.

## 3 Results

This section first presents the result of the semantic mapping between the EU bioeconomy action plan and the SDGs. Then, after the bivariate correlations amongst all identified bioeconomy related indicators are calculated, results are grouped for intra– and inter-SDGs to identify the pattern of synergies and trade-offs. Finally, the main hotspots identified at the indicator level are presented.

### 3.1 Contribution of the EU bioeconomy action plan to the SDGs

The mapping exercise depicts a wide multi-dimensionality in the scope of action of the EU bioeconomy strategy. 53 SDG targets, distributed across 12 SDGs, are mapped to one or several EU actions in the domain of the bioeconomy (Table SM2). Actions are contemplated in domains as varied as the bio-based sectors of activities (SDG 2, 8 and 9), the use and recycling of natural resources (SDG 6, 11 and 12), the biophysical environment (SDG 14 and 15), education (SDG 4), the production of bioenergy (SDG 7), climate action (SDG 13) and partnerships for the implementation of the action plan (SDG 17). Only five of the SDGs are excluded from the mapping either because they have no relation with the EU bioeconomy action plan (SDG 5: Gender equality and SDG 16: Peace, justice and strong institutions) or because their relation is not direct (SDG 1: No poverty, SDG 3: Good health and well-being and SDG 10: Reduce inequalities).

For the understanding of the following results, note that only 29 SDG targets, distributed across 11 SDGs, could be informed with data (Table SM3), after filtering for the criteria presented in section 2.1. The indicator distribution is uneven across SDGs: SDG 17 has no indicator after the filtering and is dropped; SDG 11 is represented by only one indicator (the recycling rate of municipal waste), as are SDG 13 with greenhouse gas (GHG) emissions and SDG 14 with the surface of marine sites designated under Natura 2000. The remaining SDGs are represented with 3–9 indicators. Moreover, the SDG framework defines the ‘domestic material consumption’ (DMC) as an indicator of targets 8.4 and 12.2. This duplication has been respected which statistically strengthens the synergetic relation between SDG 8 and SDG 12 all the more that the DMC is expressed in this study by five indicators for five biomass types: the DMC of wood, crops, crop residues, grazed biomass and fodder, and wild catch harvest.

### 3.2 Synergies and trade-offs between pairs of bioeconomy-related indicators

**Fig. 1:**
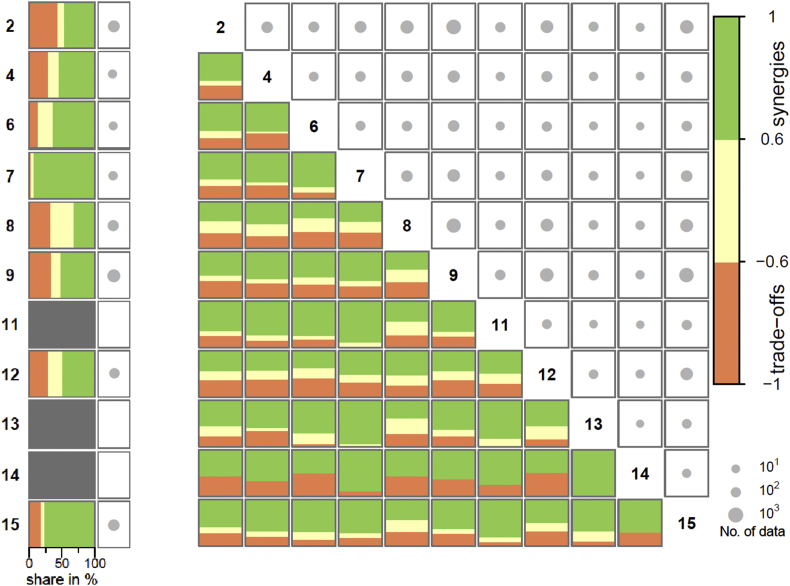
Synergies and trade-offs observed within (on the left) and between (on the right) bioeconomy-related SDGs in the EU. Note: Numbers in bold identify the SDG; “No. (Number) of data” is the number of data used for the calculation of the proportion of synergies, trade-off and non-classified correlations (i.e. pairs of time series with at least three years and significantly correlated). The figure reads: across the 28 MS, 63% of the significant correlations found between SDG 2 and SDG 4 indicators are classified as synergies (green), 28% as trade-offs (orange) and 9% were not classified (yellow). They are calculated on more than 10^2^ data pairs (medium dot) (detailed figures in Table SM4).^7^

The intra-SDG analysis applies to only eight SDGs after excluding SDG 11, SDG 13 and SDG 14 that are represented with only one indicator (see grey cells in Fig. 1, left panel), and the inter-SDG analysis has been conducted on 55 pairs of SDGs (Fig. 1, right panel). Aggregated results for the EU indicates a large predominance of synergies over trade-offs for almost all intra- and inter-SDG pairs. In 59 SDG pairs out of 63, the proportion of synergies is indeed higher than the one of trade-offs (i.e. the 59 cells of Fig. 1 where the colour green predominates). This result is consistent with the correlation analysis carried out on all SDGs by Pradhan et al. (2017) at the global level and by Muscaglia and Miola (2018) at the EU28 level.

In order to centre the analysis on the most consensual results across MS, the SDG pairs have been ranked according to the proportion of synergies and trade-offs found (left and right panels respectively, Table 1). The comparison of the top-10 synergy pair list with the top-10 trade-off pair list shows a clear distinction between SDGs aiming at a lower dependence on fossil energies (SDG 7), recycling (SDG 11) and protection of terrestrial ecosystems (SDG 15) on the one hand, and SDGs dealing with biomass production and consumption on the other hand (SDG 2, 8, 9 and 12). These results are examined in Sections 3.3, 3.4. and complemented with indicator level information.

**Table 1:**
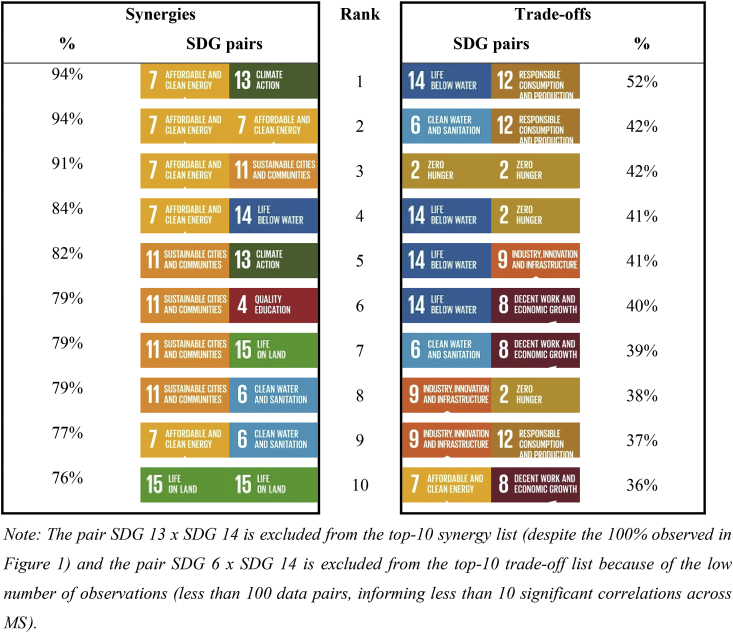
The top SDG pairs in terms of synergies (left) and trade-offs (right) of bioeconomy-related indicators in the EU.

### 3.3 Hotspots of synergies

**Fig. 2:**
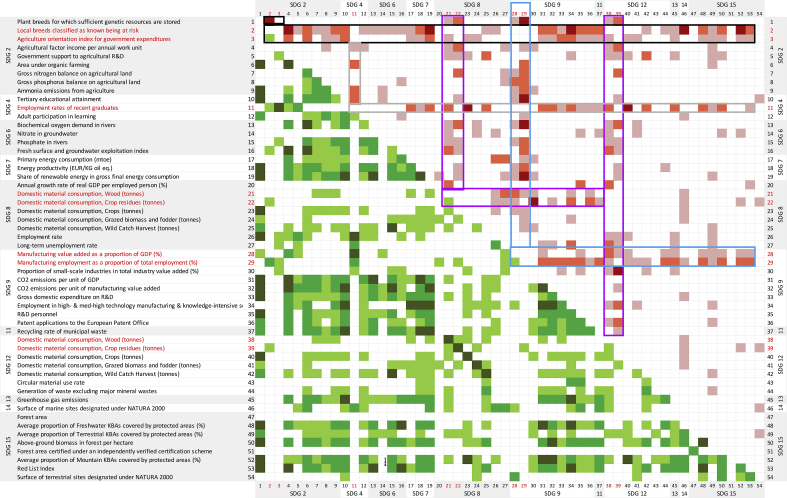
**Hotspots of synergies and trade-offs by pairs of bioeconomy-related SDG indicators** Note: To synthesise results on a single matrix, synergies between pairs of indicators are represented below the main diagonal and trade-offs above the main diagonal. Only cells where more than 50% of the pairwise correlations classified either as significant synergies (green) or trade-offs (red) and based on at least 60 data pairs are coloured. As a reminder, no causality inference can be made on this matrix, and accordingly, results have to be interpreted for the indicator pair (i and j) irrespective of whether indicator i is positioned in a row or in a column. (For interpretation of the references to colour in this figure legend, the reader is referred to the Web version of this article.) Legend: dark green (red) cells indicate indicator pairs with 100% synergies (trade-offs), medium green (red) indicate 75%–100% synergies (trade-offs), light green (red) cells indicate 50%–75% synergies (trade-offs). For example, the dark red cell corresponding to indicator 1 in row and indicator 2 in column indicates 100% synergies found between those two indicators. Cells highlighted with coloured frames represent indicators (marked in red) where systematically trade-offs are found (i.e. hotspots) (e.g. in purple, two indicators common to SDG 8 and SDG 12; in black, two indicators related to SDG 2).

The top-10 synergy pair list is dominated by SDG 7 (Clean energy) and SDG 11 (municipal recycling) with six and five occurrences respectively (Table 1, left). Both of them appear strongly positively correlated with SDG 6 (Clean water) and SDG 13 (GHG emissions), suggesting that any improvement towards one of these four bioeconomy-related SDGs will be correlated with progress towards the other three. SDG 7 indicators also correlate positively between themselves in 94% of the significant correlations found (SDG 7 x SDG 7) and with the ‘surface of marine sites designated under Natura 2000’ (SDG 14) in 84% of the data pairs (Table 1, left). Fig. 2 shows another hotspot of synergies between SDG 7 indicators and those SDG 9 indicators related to innovation and CO_2_ emissions (see medium and dark green cells for indicators 17–19 x indicators 31–36 in Fig. 2 that indicate 75%–100% synergies on significant correlations calculated on more than 60 data pairs). Similarly, the strong synergy found between SDG 11 (municipal recycling) and SDG 4 (Education) is largely due to the relation of municipal recycling with tertiary educational attainment for which all the 24 significant correlations found are positive (see dark green cell for indicator 10 x indicator 37 in Fig. 2).

SDG 15 (Life on land) ranks three times in the top-10 synergy pair list: at the 5th position in association with SDG 11 (municipal recycling) and at the 10th position with itself (SDG 15 x SDG 15) (Table 1, left). Intra-SDG 15 synergies (76% of the data pairs, Table 1) are largely due to a strong homogeneity of the eight SDG 15 indicators. Apart from forest area and forestry biomass yield indicators, they all measure progress in the protection of biodiversity (i.e. Red List index) or ecosystems (i.e. protected freshwater, terrestrial and mountain Key Biodiversity Areas (KBAs), terrestrial sites under Natura 2000, certified forests).

### 3.4 Hotspots of trade-offs

The top-10 trade-off pair list is shared between SDGs related with agricultural production and agriculture impacts (SDG 2), industrial use of biomass (SDG 9) or with the measure of domestic material consumption (SDG 8 and 12) (three or four occurrences each). Surprisingly, although SDG 14 and SDG 15 share similar aims - the protection of marine and terrestrial ecosystems respectively – SDG 14 ranks four times in the top-10 trade-off pair list (Table 1, right) while SDG 15 is well represented in the top-10 synergy list (Table 1, left).

Moving on to the trade-off list, it is interesting to highlight that only in two of the SDG pairs the proportion of trade-offs actually dominate over synergies. These are: SDG 12 (Biomass consumption and production) x SDG 14 (marine Natura 2000 sites) (52% vs. 48% of significant correlations) and SDG 6 (Clean water) x SDG 8 (Industry, innovation and Infrastructure) (39% vs. 35% of significant correlations) (see Fig. 1). In other words, even amongst those pairs of SDGs where trade-offs are encountered more frequently, still synergies predominate over trade-offs (with the two exceptions commented).

Therefore, the trade-off analysis makes more sense at the indicator level as shown in Fig. 2. This figure highlights only the indicator pairs for which a proportion of significant synergies or trade-offs higher than 50% has been found over a sample of more than 60 data pairs. While the majority of the indicator pairs are characterised with synergies (green cells), four clear hotspots of trade-offs emerge on the heat map (see colour frames around red cells). They correspond to a limited number of indicators that are negatively correlated with almost the whole range of other bioeconomy-related SDG indicators of this study:

- Trade-offs related to SDG 2 indicators (Fig. 2, black frame) on agro-biodiversity (i.e. the number of local breeds classified as known being at risk) and the agriculture orientation index for government expenditures.
- Trade-offs related to two SDG 9 indicators (Fig. 2, blue frame) on the weight of manufacturing sectors in terms of value-added and jobs.
- Trade-offs related to the same two SDG 8 and SDG 12 indicators (Fig. 2, purple frame) on DMC of wood and crop residues.

The trade-offs arising from the employment rate of recent graduates (SDG 4, Fig. 2, grey frame) will not be commented further as SDG 4 does not appear on the top-10 trade-off list (Table 1, right).

## 4 Discussion

Section 3 presented the findings of the study in answer to the two research questions. The present section discusses these findings in the context of the literature (sections 4.2. to 4.7.) after recognising the limitations and strengths of the methodological approach (section 4.1). Strong emphasis is placed at examining if the correlations underlying the main areas of synergies and trade-offs highlighted in the results section are supported by empirical observations and scientific studies. Furthermore, the discussion examines whether those correlations play or have played a role in the design of the EU policy framework.

### 4.1 Strengths and limitations of data and methodology

This study draws on the methodological approach proposed by Pradhan et al. (2017) for the identification of synergies and trade-offs amongst SDGs and applies this approach to the field of EU policy coherence between bioeconomy actions and the SDGs achievements. In comparison with Pradhan et al. (2017), the application field has constrained the statistical analysis to a fewer number of countries (only the EU MS) and SDGs (only those bio-economy-related, see section 2.1), whilst the elimination of redundant SDG indicators has further reduced the database (see section 2.1). Nevertheless, the European scope of the study has also allowed the enrichment of the database with additional Eurostat data on specific bio-economy-related SDG indicators.

As a note of caution, it is necessary to acknowledge the limitations of the study. One important data limitation lies in that neither the UN nor Eurostat covers all the identified bioeconomy-related SDG targets. Indicator incompleteness in terms of years and Member State representation, together with requirements in data harmonization, have also exacerbated the over-/under-representation of some SDGs and may have created bias in the identification of the reinforcing or restricting bioeconomy-related forces in play. Moreover, SDG indicators are calculated at the national level but do not capture extra-borders impacts. This is an important weakness of this study since the extra-EU impacts of the European bioeconomy are not negligible.

At the methodological level, the paper opted for a fully quantitative analysis. Compared to other qualitative or semi-quantitative approaches, the procedure can be replicated for other time and geographical samples (e.g. other bioeconomy concepts in use in other countries or regions). The reliance on quantitative output also reduces the subjectivity of the results even though, as mentioned earlier, conclusions are tied to data availability and quality. The magnitude of the correlation coefficients and the proportion of positive or negative correlations inform on the strength of the association, but no indication is given on the direction of causality. Finally, the ex-post analysis captures synergies and trade-offs under past conditions that do not necessarily reflect the structural dynamics of the future socio-economic system.

### 4.2 SDG 7: the biofuel case and the evolution of the EU Renewable Energy Directive

In the bioeconomy context, the high synergetic profile of SDG 7 (Clean energy) has to be examined from the perspective of bioenergies (i.e. 65% of the EU renewable energies in volume (Eurostat, 2018b)). In fact, almost nothing is found in the scientific literature on their relation with municipal recycling (SDG 11) or maritime protected areas (SDG 14). However, it appears that the specificities of biomass as a feedstock for energy production could, in fact, compromise the correlation between bioenergies and energy productivity/efficiency, between bioenergies and water quality and utilization, and with GHG emissions.

In their literature review, Ji and Long (2016) enumerate multiple concerns on the impact of all types of biofuels on water quality and water usage during the production and processing stages. However, the magnitude of these effects varies according to the processing technology and to the region (especially when irrigation water is mobilised) (Wu et al., 2009). The positive sign or negative relationship between biofuels and energy productivity and between biofuels and GHG emissions depends on the biofuel generation (crop vs. lingo-cellulosic material vs. algae vs. waste), the production system (e.g. natural conditions, fertilisation, irrigation, technologies…) and the method of calculation, even without accounting for indirect land-use change (iLUC) effects (de Gorter et al., 2013; Hennecke et al., 2013; Lewandowski, 2015; Ji and Long, 2016; García-Condado et al., 2019). In the case of the present study, it is important to remind that correlations are run on indicators measured in EU MS and do not reflect extra-EU effects (e.g. trade and iLUC effects).

Interestingly, scientific debates on the relation between biofuel consumption, energy efficiency and GHG emission have been reflected in the legislative process with consecutive amendments to the Renewable Energy Directive (Dir. (EU) 2015/1513 and then in Dir. (EU) 2018/2001 (European Union, 2015; European Union, 2018b)). A biofuel transition towards those biofuels with higher potential of GHG emission saving and lower iLUC effect was indeed encouraged with the introduction of specific targets for “advanced” biofuels and sustainability criteria for biofuel accounting in national targets. Water quality and water utilization are not part of the sustainability criteria but are included in several voluntary schemes in place in MS.

### 4.3 SDG 11: the co-benefits of recycling targeted by the EU waste framework directive

The synergetic profile of municipal recycling found in the present study is confirmed in the following scientific studies. The labour-intensity of recycling activities and their potential for productivity increase are supposed to offer economic and job opportunities in the waste management sector (SDG 8), while the skill adjustments needed for their upscale is also expected to steer education and vocational training (SDG 4) (Schroeder et al., 2019). The relation between waste recycling and SDG 7 (Clean energy) is materialised by the annual production of 676 PJ of energy from waste in the EU (Saveyn et al., 2016). Waste-to-energy processes permit GHG emission savings (SDG 13) from the avoided methane emissions from landfill, and thanks to lower CO_2_ emissions per MWh in waste-to-energy power plants than in fossil fuel power plants (AlQattan et al., 2018). They also allow for fertilizer production from waste’s nutrients (EBA, 2015). In addition, recycling permits to reduce environmental pollution (SDG 6) from landfilling, to reduce raw material demand and to improve resource efficiency (SDG 8 and SDG 12) (Eurostat, 2018c). However, Mayer et al. (2019) stress particular cases in which recycling indirectly required more material or energy than the direct use of primary materials.

At the policy level, the expectation of co-benefits (or synergies) from waste reuse and recycling have motivated the elaboration of the Circular Economy package (European Commission, 2015). In particular, this package includes the recent setting of municipal waste recycling targets in the directive (EU) 2018/851 (European Union, 2018a) and the on-going discussion on the revision of the Fertilizing Products legislation. The latter aims at easing and fostering the production of organic fertilisers manufactured from secondary raw materials such as agricultural by-products and recovered bio-waste.

### 4.4 SDG 15: area protection and biodiversity protection, rationale of the Natura 2000 network

In the present correlation analysis, intra-SDG 15 synergies mainly relate to positive correlations between area protection and biodiversity status. This relationship underlies most policy strategies for biodiversity (see EU MS commitments for Target 1 of the EU 2020 biodiversity strategy (European Commission, 2011a), the UN-SDG target 15 and the Convention on Biological Diversity “Aichi-target” 11 (CBD, 2010)). At the legislative level, the Nature directives have motivated the implementation of the Natura 2000 network in the EU that has become the largest coordinated network of protected areas in the world (European Union, 1992; 2009). This network is found to be beneficial for the biodiversity of common species and species at risk (Jones-Walters et al., 2016) and to have a stabilizing and preventing role from further biodiversity decline (EEA, 2015). Though, leveraging the synergy between area protection and biodiversity status might not be sufficient as more than 50% of the species identified at risk show an unfavourable status (EEA, 2015). A wider ecological representation and better management effectiveness of protected areas are the main proposals for improvement (Watson et al., 2014; Friedrichs et al., 2018; Lemieux et al., 2019), and particularly when freshwater biodiversity is concerned (Juffe-Bignoli et al., 2016; Carrizo et al., 2017; Bastin et al., 2019). Future climate conditions might also trigger changes in species distribution and imply the revision of the geographical coverage of the Natura 2000 network (Araújo et al., 2011; Watson et al., 2014; Blicharska et al., 2016; Juffe-Bignoli et al., 2016; Carlson et al., 2017; Friedrichs et al., 2018). Moreover, the last biodiversity assessment for Europe concludes that a wider range of policies than area protection measures are needed to address direct and indirect drivers of biodiversity loss, the main ones being land-use change, the impacts of climate change, increasing natural resource extraction, pollution and invasive alien species (IPBES, 2018).

### 4.5 SDG 2: the difficult challenge of curbing the agriculture – agro-biodiveristy trade-off

Trade-offs with SDG 2 indicators mainly relate to agro-biodiversity and the agriculture orientation of an MS. In the scientific literature, the erosion of agro-biodiversity is directly attributed to the large adoption of new highly productive but little diversified genetic material (FAO, 2010, FAO, 2015; Govindaraj et al., 2015; UNEP/, 2016/UNECE, 2012; Bioversity International, 2017), the implementation of monocultures and the use of pesticides. Other indirect factors are mentioned like over-exploitation (e.g. over-grazing) and land cover changes (Govindaraj et al., 2015). Nitrogen and Phosphorus pollution from inefficient management of manure and the use of synthetic fertilizers also affect the capacity of plants and animals to survive (Guthrie et al., 2018; Kanter and Brownlie, 2019).

Political concerns for curbing the negative effects of agriculture on agro-biodiversity have been materialised by MS efforts for ex-situ conservation in gene banks (FAO, 2010) as well as the introduction of greening measures in the EU Common Agriculture policy in 2010 and 2013, that is, crop diversification, maintenance of permanent grassland and Ecological Focus Areas (EFAs) (Hodge et al., 2015; Tzilivakis et al., 2016). However, as posited by Pe’er et al. (2014), these efforts might not meet the agro-biodiversity challenge.

Nutrient pollution from agricultural origin is addressed by several policy instruments: the EU water framework directive, the Nitrates directive (European Union, 1991; 2000) and the Gothenburg protocol on ammonia (NH3) volatilisation and nitrogen oxides (NOx) emissions (UNECE, 2012). The volatilisation of nitrogen dioxide (N2O) of agriculture origin is not regulated. However, the eight infringement cases to the Nitrate directive give an indication of the difficulty for the MSs to comply with these directives (European Commission, 2018a).

### 4.6 SDG 8 and 12: the challenge of decoupling biomass DMC from bioeconomy activities

Trade-offs related to SDG 8 and SDG 12 indicators mainly relate to the DMC of wood and crop residues. They should be given special attention since the development of the bioeconomy could further exacerbate the consumption of biomass for the production of bio-based products and because over-consumption of biomass was among the points of concern raised after the release of the first EU bioeconomy strategy (see section 1). The coupling of natural resources consumption and environmental degradation was initially pointed in 1987 in the Brundtland report (WCED, 1987). Since then, miscellaneous scientific studies have confirmed the existence of trade-offs between the occidental consumption level and the status of the living environment. The purpose of the present section is not to enumerate them, but rather to observe whether this knowledge plays or has played a role in the design of EU policies.

The 2011 Roadmap for a resource efficient Europe envisaged measures in the fields of research and innovation (R&I), consumer information on the environmental footprint of marketed products and waste management for better re-use and recycling (European Commission, 2011b). Waste and secondary material management proposals have been integrated into the EU Circular Economy package (European Commission, 2015) while consumer information (including labels and certifications) and R&I supports are part of the EU bioeconomy action plan (European Commission, 2018b). Concerning the use of biomass for energy, woody biomass has to meet sustainability criteria and obligations defined by the EU (European Union, 2010; 2015) but no criteria apply so far to crop residues.

The bioeconomy is a relatively new concept and it has not been subject to any integrated impact assessment so far. However, the development of footprint quantifications is likely to better inform on the biomass or the GHG footprint of bioeconomy activities in the near future.

### 4.7 SDG 9: the challenge of ensuring sustainable bio-based manufacturing sectors

Trade-offs related to SDG 9 indicators mainly relate to industrial development (measured in terms of proportion of value-added and jobs in the manufacturing sectors). These points of conflict cannot be directly transposed to the development of bio-based industries since they represent a small subset of the industrial sector, i.e. 27% of jobs and 22% of value-added of total EU28 industry in 2015 (Eurostat, 2018a; Ronzon et al., 2018). Bioeconomy promotors claim instead that the development of the bioeconomy will create new markets for agricultural commodities and diversify rural economies. Mengal et al. (2018) expect bio-based industries to boost employment, 80% of which taking place in rural and underdeveloped areas. On the environmental side, new bio-based products would have the potential to save GHG emissions compared to their fossil counterpart (Carus, 2017; Mengal et al., 2018). The mobilization of waste and the cascading and circular uses of biomass in bio-based value-chains would boost biomass productivity compared to the past, thereby lessening the tension between industrial activity and biomass DMC (Bell et al., 2018; Mengal et al., 2018).

Although new bio-based products have recently emerged and advanced technologies are being tested in pilot plants, the scaling up of bio-based industries has not materialised yet and it is difficult to assess the relation between bio-based industry development and the SDGs. Coherently with agricultural, industrial and environmental EU policies, the challenge of the new EU bioeconomy action plan is exactly to address past trade-offs between industry and agriculture and natural resources and ecosystems.

## 5 Conclusions

Relying on a quantitative analysis of pair-wise correlations between bioeconomy-related SDG indicators, the paper aims at (i) identifying relevant inter-linkages that later on could inform ex-ante simulation models in terms of SDGs characterisation and impacts and (ii) providing data-based evidence in support of future EU policy coherence.

Regarding the first aim, this study stresses the strong inter-linkages of some common modelling indicators with a wider range of other SDG indicators. The hotspot of trade-offs related to SDG 9 indicators is a good illustration. Examining correlations observed between the indicators ‘proportion of manufacturing value added in total GDP′ and the ‘proportion of manufacturing jobs in total jobs’, with other non-market SDG indicators, reveals a series of trade-offs. A more advanced econometric analysis could help parametrise such market-nonmarket relationships. Their integration within a price-driven simulation modelling framework, in a second step, would then permit ex-ante assessments of the performance of the selected non-market indicators according to the development of the two SDG 9 indicators above-mentioned under different scenario designs.

Regarding the second aim, the methodology tested in this study can be seen as a screening method to identify important points of synergy and conflict associated with a given policy action plan. Provided data is available, the methodology can be replicated at other national or regional levels to account for different socio-economic and environmental contexts. Future updates are also possible to assess progress at strengthening synergies and addressing trade-offs. The *ex-post* analysis conducted in this paper shows a high representation of environment-related SDGs in the top 10 synergies pair list: SDG 6 (water), SDG 7 (biofuels), SDG 11 (recycling), SDG 13 (climate action) and SDGs 14 and 15 (aquatic and terrestrial ecosystems). Actions strengthening environmental sustainability thus appear as key enabling forces for the achievement of multiple SDGs related to all three sustainability dimensions. On the other hand, mixed results are found regarding the social and economic pillars of sustainable development. To mention a few examples, indicators of value-added (economic dimension) and employment (social dimension) in the manufacturing sector constitute a hotspot of trade-offs while a synergetic profile is observed from the indicators of the gross domestic expenditure on research and development (economic dimension) or the tertiary educational attainment (social dimension).

Overall, we find that synergetic developments by far dominate over trade-offs among and within bioeconomy-related SDGs. Across the 28 EU MSs, improvements towards energy efficiency and the development of renewable energies (SDG 7), efforts in municipal recycling (SDG 11) and preservation of ecosystems and their biodiversity (SDG 15) have been concomitant to progress towards many other bioeconomy-related targets. The maintenance and improvement of the wide range of EU policy instruments in place in these domains is critical since they can bring many co-benefits. On the other hand, the withdrawal of policy instruments might stop progress or even entail the degradation not only of the targeted indicator but also of other correlated ones. This network of correlations might then accentuate the cost of political inaction.

The discussion highlights that the main domains of trade-offs have most of the time already been identified, triggering the implementation of EU policy instruments. These findings call for more efficient and coherent policy actions. But given the difficulty to overcome trade-offs over the period observed (1990–2018), a broader array of actions might be needed than the only policy ones (e.g. corporate responsibility, consumer awareness, organisational and cultural changes). The trade-offs identified in relation with SDG 2 and SDG 9 call for a change in agricultural and industrial production processes in such a way that they place less pressure on the environment (e.g. water, biodiversity and ecosystems represented in SDG 2, SDG 6, SDG 14 and SDG 15). Change in practices, technical and technological innovations (Wesseler and von Braun, 2017) and the application of circular and ‘cascading principles’ are the most common suggestions on the table (European Commission, 2015). But trade-offs associated with Europeans’ consumption of biomass materials (SDG 8 and SDG 12) call for much more than technical solutions.

## Funding

This project has received funding from the European Union’s Horizon 2020 research and innovation programme under grant agreement No 773297 and from the European Commission’s Administrative Arrangement N° JRC 34488-2016.

## Declaration of competing interest

The authors declare that they have no known competing financial interests or personal relationships that could have appeared to influence the work reported in this paper. The views expressed are those solely of the authors and should not in any circumstances be regarded as stating an official position of the European Commission.

## Footnotes

1 This broad definition covers a multitude of variants, two of which are formulated in the two successive EU bioeconomy strategies launched by the EU: (i) the bioeconomy ‘encompasses the production of renewable biological resources and the conversion of these resources and waste streams into value added products, such as food, feed, bio-based products and bioenergy’ (European Commission, 2012), and (ii) the bioeconomy ‘includes and interlinks: land and marine ecosystems and the services they provide; all primary production sectors that use and produce biological resources (…); and all economic and industrial sectors that use biological resources and processes to produce food, feed, bio-based products, energy and services’ (European Commission, 2018b).

2 Market-based indicators are those metrics that can be provided by market-driven price-based simulation modelling frameworks (e.g. manufacturing value added as a proportion of gross domestic product (GDP) in SDG 9), as opposed to non-market indicators (e.g. R&D personnel for SDG 9).

3 For instance, the sub-action 1.1 ″Mobilise public and private stakeholders, in research, demonstration and deployment of sustainable, inclusive and circular bio-based solutions” relates to the matching field “Investment, funding and Public-Private Partnerships (PPPs)" and with the UN SDG target 17.17 that “encourages and promote effective public, public-private and civil society partnerships (…)".

4 This is an arbitrary selection (as in Pradhan et al., 2017) that aims at minimizing the loss of indicators due to incompleteness of time series.

5 For example, related to the SDG target 15.a on financial resources to conserve and sustainably use biodiversity and ecosystems, the indicator 15.a.1 only measures “official development assistance” to developing countries. This indicator does not reflect any field of action of the EU Bioeconomy strategy and has been discarded.

6 An example is the Eurostat indicator sdg_06_60 that measures the degree of water exploitation. Fresh ground water and fresh surface water data are excluded and only the data related with the exploitation of fresh water as a total of the former two are kept.

7 We thank P. Pradhan for providing the R-code used for the graphical depiction of results in this figure.
