# Supplementary material for: Friends or foes? A compatibility assessment of bioeconomy-related Sustainable Development Goals for European policy coherence
Source: J Clean Prod. 2020 May 1;254:119832. doi: 10.1016/j.jclepro.2019.119832 (PMC7171703; doi:10.1016/j.jclepro.2019.119832)
Supplement: Multimedia component 1 [file mmc1.docx]

**Table SM1 – Match between Action plan, matching fields and the targets of the sustainable development goals (SDGs)**

| **Bioeconomy actions** | **Matching fields** | **Associated UN SDG targets** |
| --- | --- | --- |
| 1.1, 1.2, 1.6, 2.1, 2.2 | Investment/funding/public-private partnership (PPP) in the bioeconomy | 2.3; 2.a; 7.a; 8.3; 9.3; 15.a; 15.b; 17.17 |
| 2.1, 2.4 | Education-awareness/training/skills in the bioeconomy | 4.4; 4.7; 12.8 |
| 1.1, 1.3, 1.6, 2.1, 2.2, 3.1, 3.2 | Research/Innovation/measurement in the bioeconomy | 2.a; 7.a; 8.2; 8.3; 9.5; 9.b; 14.a; 17.19 |
| 1.1, 1.2, 1.4, 1.5, 2.1, | Technological upgrade – Demonstration in the bioeconomy | 2.a; 7.a; 7.b; 8.2; 9.4; 9.5; 9.b |
| 1.3, 1.4,.2.1, 2.2, 2.3, 3.4 | Policy and market incentives, policy planning and awareness | 11.a; 11.b; 12.6; 12.7; 15.9; 17.14 |
| 1.1, 1.2, 1.4, 1.5, 2.1, 2.2, 3.4 | Sustainable production/harvesting of biomass | 2.3; 2.4; 14.7; 14.b; 15.1; |
| 1.1, 1.2, 1.4, 1.5, 2.1 | Sustainable conversion of biomass/BBI | 7.2; 9.2 |
| 1.1, 1.2, 1.4, 1.5, 2.1, 2.2 | Circular/Resource-efficient production/waste reduction and management/biorefineries | 6.4; 6.a; 7.3; 8.4; 9.4; 11.6; 12.3; 12.5 |
| 1.4, 1.5,1.6, 2.1, 2.2, 3.1, 3.3 | Sustainable management of natural resources and ecosystems | 6.3; 6.4; 6.5; 12.2; 14.1; 14.2; 14.7; 15.1; 15.2 15.a; 15.b |
| 3.1, 3.2, 3.3, 3.4 | Conservation, biodiversity | 2.5; 2.a; 15.1; 15.4; 15.5; 15.a; 15.b |
| 1.6, 2.2, 3.2, 3.3 | Restoration, bio-remediation | 6.6; 14.2; 14.3; 14.4; 15.1; 15.2; 15.3 |
| 1.2, 1.4, 1.5, 3.2 | Climate action | 13.2; 13.3 |
| 1.2, 1.5, 2.1, 2.2 | Local development (jobs and growth) | 2.3; 2.a; 8.2; 8.3; 8.5; 9.2; 9.3; 14.b |

**Table SM2 – Match between actions of the bioeconomy action plan and SDG targets per SDG**

| Bioeconomy actions | SDG targets |
| --- | --- |
| **SDG 2 (End hunger)** |  |
| action 1.1 - Public-private partnerships  action 1.2 - Circular Economy Thematic Investment Platform  action 1.4 - Development of environmental labels and standards  action 1.5 - Development of integrated biorefineries  action 2.1 - National and regional Strategic Deployment Agendas (SDAs)  action 2.2 - Pilot actions for a local bioeconomy development  actions 3.1 to 3.3 - Data measurement and monitoring  action 3.4 - Roadmap to agro-ecology | target 2.3 - "double the agricultural productivity and incomes of small-scale food producers (...), including through access to (…) financial services"  target 2.4 - "ensure sustainable food production systems, (…) that help maintain ecosystems"  target 2.5 - "maintain the genetic diversity of seeds, cultivated plants and farmed and domesticated animals and their related wild species"  target 2.a - "Increase investment (…) in rural infrastructure, agricultural research and extension services, technology development" |
| **SDG 4 (Quality education)** |  |
| action 3.2.4. - Promote education, training and skills across the bioeconomy | target 4.4 - "increase the number of youth and adults who have relevant skills"  target 4.7 - "ensure that all learners acquire the knowledge and skills needed to promote sustainable development" |
| **SDG 6 (Clean water)** |  |
| action 1.4 - Development of standards and labels  action 1.5 - Development of integrated biorefineries  action 2.1 - National and regional Strategic Deployment Agendas  action 2.2 - Urban Circular Bioeconomy Strategies and "living labs"  action 3.2 and action 3.3 - Measurement, monitoring and guidance activities | target 6.3 - "improve water quality by reducing pollution"  target 6.4 - "increase water-use efficiency across all sectors"  target 6.5 - "implement integrated water resources management"  target 6.6 - "protect and restore water-related ecosystems"  target 6.a - "including (…) water efficiency, wastewater treatment, recycling and reuse technologies" |
| **SDG 7 (Clean energy)** |  |
| action 1.1 - Investment from public-private partnerships  action 1.2 - Circular Economy Thematic Investment Platform  action 1.4 - Development of labels and standards  action 1.5 - Development of integrated biorefineries  action 2.1 - Research and technological upgrade foreseen in Strategic Deployment Agendas | target 7.2 - "increase substantially the share of renewable energy in the global energy mix"  target 7.3 - "improvement in energy efficiency"  target 7.a - "access to clean energy research and technology" and "promote investment in (…) clean energy technology"  target 7.b - "upgrade technology for supplying modern and sustainable energy services" |
| **SDG 8 (Decent work and economic growth) and SDG 9 (Industry, innovation and infrastructure)** | |
| action 1.1 - Public-private partnerships  action 1.2 - Circular Economy Thematic Investment Platform  action 1.3 - Guidance to the deployment of bio-based innovations  action 1.4 - Development of labels and standards  action 1.5 - Development of integrated biorefineries  action 1.6 - R&I investments for the development of substitutes to fossil based materials  action 2.1 - National and regional Strategic Deployment Agendas  action 2.2 - Pilot actions for a local bioeconomy development | target 8.2 - "higher levels of economic productivity through diversification, technological upgrading and innovation (…)"  target 8.3 - "support productive activities (…), creativity and innovation, and encourage the formalization and growth of micro-, small- and medium-sized enterprises"  target 8.4 - "Improve (…) resource efficiency in consumption and production"  target 8.5 - "achieve full and productive employment"  target 9.2 - "Promote inclusive and sustainable industrialization (…), significantly raise industry’s share of employment and gross domestic product"  target 9.3 - "Increase the access of small-scale industrial (…)to financial services (…) and their integration into value chains and markets"  target 9.4 - "retrofit industries to make them sustainable, with increased resource-use efficiency and greater adoption of clean and environmentally sound technologies and industrial processes"  target 9.5 - "Enhance scientific research, upgrade the technological capabilities of industrial sectors"  target 9.b - "Support domestic technology development, research and innovation (…) for, inter alia, industrial diversification" |
| **SDG 11 (Sustainable cities and communities)** |  |
| action 2.1 - National and regional Strategic Deployment Agendas  action 2.2 - Sea basin strategies, Urban Circular Bioeconomy Strategies and living labs  action 2.3 - European Bioeconomy policy support facility | target 11.6 - " by paying special attention to (…) municipal and other waste management"  target 11.a - "by strengthening national and regional development planning"  target 11.b - "implementing integrated policies and plans towards inclusion, resource efficiency, mitigation and adaptation to climate change" |
| **SDG 12 (Responsible consumption and production)** |  |
| actions 1.1 and 3.1.2 – Investments  action 1.4 - Development of environmental labels and standards  action 1.5 - Development of integrated biorefineries  action 1.6 - Research and innovation  action 2.1 - National and regional Strategic Deployment Agendas  action 2.2 - Pilot actions for a local bioeconomy development  action 2.4 - Education  action 3.1 - Enhance the knowledge on the bioeconomy (…), and make it accessible  actions 1.3, 2.3 and 3.3 - Guidance  action 3.4 (iii) - The private sector (…) should be encouraged to use the information (…) | target 12.2 - "sustainable management and efficient use of natural resources"  target 12.3 - "reduce food losses along production and supply chains"  target 12.5 - "reduce waste generation through prevention, reduction, recycling and reuse"  target 12.6 - "Encourage companies (…) to adopt sustainable practices"  target 12.7 - "Promote public procurement practices that are sustainable"  target 12.8 - "ensure that people everywhere have the relevant information and awareness for sustainable development" |
| **SDG 13 (Climate action)** |  |
| action 1.2 - Circular Economy Thematic Investment Platform  action 1.4 - Deployment of standards and labels on the basis of data on climate performance  action 1.5 - Development of integrated biorefineries  action 3.2 - Bioeconomy monitoring system | target 13.2 - "Integrate climate change measures"  target 13.3 - "institutional capacity on climate change mitigation, adaptation, impact reduction" |
| **SDG 14 (Life below water)** |  |
| action 1.1 to 3.1.6 - Investments, research and innovation, markets [in particular action 1.6 - free plastic oceans]  action 2.1 - national and regional Strategic Deployment Agendas (SDAs)  action 2.2 - development of Sea Basin Strategies, pilot actions to the Blue Bioeconomy potential  actions 3.2 and 3.3 - observation, measurement and monitoring | target 14.1 - "reduce marine pollution"  target 14.2 - "sustainably manage and protect marine and coastal ecosystems" and "take action for their restoration"  target 14.3 - "Minimize and address the impacts of ocean acidification"  target 14.4 - "science-based management plans, in order to restore fish stocks"  target 14.7 - "sustainable management of fisheries, aquaculture'  target 14.a - "Increase scientific knowledge, develop research capacity and transfer marine technology"  target 14.b - "access for small-scale artisanal fishers to marine resources and markets" |
| **SDG 15 (Life on land)** |  |
| action 1.1 - public-private partnerships  actions 1.1, 1.2 and 1.6 - investments for land ecosystem management, conservation and restoration/bioremediation  action 1.4 - Deployment of standards and labels on the basis of data on environmental and climate performance  action 1.5 - development of integrated biorefineries  action 2.1 - National and regional Strategic Deployment Agendas (SDAs)  action 2.2 - Rehabilitation of urban brownfields, nature-based remediation solutions  action 3.1 - Enhance the knowledge on biodiversity and ecosystems, and make it accessible  action 3.2 - Monitoring system to underpin ecosystem conservation and restoration  3.3 - Guidance on integrating ecosystem services into decision making | target 15.1 - "ensure the (…) sustainable use of terrestrial and inland freshwater ecosystems and their services"  target 15.2 - "sustainable management of all types of forests, (…) restore degraded forests and substantially increase afforestation and reforestation"  target 15.3 - "restore degraded land and soil"  target 15.4 - "conservation of mountain ecosystems, including their biodiversity"  target 15.5 - "protect and prevent the extinction of threatened species"  target 15.9 - "integrate ecosystem and biodiversity values into national and local planning"  target 15.a - "increase financial resources (…) to conserve and sustainably use biodiversity and ecosystems"  target 15.b - "finance sustainable forest management (…), including for conservation and reforestation" |
| **SDG 17 (Partnerships for the goals – Policy and institutional coherence)** |  |
| actions 1.1, 2.1 and 2.2 - Policy coherence for sustainable development  actions 1.3, 2.1, 2.3 and 3.1 - Public-private and society partnerships  actions 3.1 and 3.2 - Measurement of progress on sustainable development | target 17.14 - "policy coherence for sustainable development"  target 17.17 - "effective public, public-private and civil society partnerships"  target 17.19 - "develop measurements of progress on sustainable development", "support statistical capacity-building" |

**Table SM3- Characteristics of the bioeconomy related SDG indicators used in the correlation analysis**

|  | **SDG** | **Target** | **Indicator** | **Source** | **Sign** | **No.MS** | **No. of data points** | **Minimum year** | **Maximum year** |
| --- | --- | --- | --- | --- | --- | --- | --- | --- | --- |
| 1 | 2 | 2.5 | Plant breeds for which sufficient genetic resources are stored (number) | UN | 1 | 26 | 208 | 1995 | 2017 |
| 2 | 2 | 2.5 | Local breeds classified as known being at risk (number) | UN | -1 | 28 | 786 | 1990 | 2018 |
| 3 | 2 | 2.a | Agriculture orientation index for government expenditures | UN | 1 | 26 | 372 | 2001 | 2016 |
| 4 | 2 | 2.3 | Agricultural factor income per annual work unit (AWU) (source: EC services) | Eurostat | 1 | 28 | 504 | 2001 | 2018 |
| 5 | 2 | 2.a | Government support to agricultural research and development | Eurostat | 1 | 28 | 420 | 2004 | 2018 |
| 6 | 2 | 2.4 | Area under organic farming | Eurostat | 1 | 28 | 504 | 2000 | 2017 |
| 7 | 2 | 2.4 | N - Gross nutrient balance on agricultural land by nutrient | Eurostat | -1 | 28 | 476 | 2000 | 2016 |
| 8 | 2 | 2.4 | P - Gross nutrient balance on agricultural land by nutrient | Eurostat | -1 | 28 | 476 | 2000 | 2016 |
| 9 | 2 | 2.4 | Ammonia emissions from agriculture (source: EEA) | Eurostat | -1 | 28 | 756 | 1990 | 2016 |
| 10 | 4 | 4.4 | Tertiary educational attainment | Eurostat | 1 | 28 | 504 | 2000 | 2017 |
| 11 | 4 | 4.7 | Employment rates of recent graduates | Eurostat | 1 | 28 | 336 | 2006 | 2017 |
| 12 | 4 | 4.7 | Adult participation in learning | Eurostat | 1 | 28 | 504 | 2000 | 2017 |
| 13 | 6 | 6.3 | Biochemical oxygen demand in rivers (source: EEA) | Eurostat | -1 | 20 | 300 | 2000 | 2014 |
| 14 | 6 | 6.3 | Nitrate in groundwater (source: EEA) | Eurostat | -1 | 21 | 273 | 2000 | 2012 |
| 15 | 6 | 6.3 | Phosphate in rivers (source: EEA) | Eurostat | -1 | 20 | 300 | 2000 | 2014 |
| 16 | 6 | 6.4 | Fresh surface and groundwater exploitation index | Eurostat | -1 | 25 | 400 | 2000 | 2015 |
| 17 | 7 | 7.a | Primary energy consumption (mtoe) | Eurostat | -1 | 28 | 476 | 2000 | 2016 |
| 18 | 7 | 7.b | Energy productivity (EUR/KG oil eq.) | Eurostat | 1 | 28 | 476 | 2000 | 2016 |
| 19 | 7 | 7.2 | Share of renewable energy in gross final energy consumption by sector | Eurostat | 1 | 28 | 364 | 2004 | 2016 |
| 20 | 8 | 8.2 | Annual growth rate of real GDP per employed person (%) | UN | 1 | 28 | 504 | 2000 | 2017 |
| 21 | 8 | 8.4 | Domestic material consumption, Wood (tonnes) | UN | -1 | 28 | 491 | 2000 | 2017 |
| 22 | 8 | 8.4 | Domestic material consumption, Crop residues (tonnes) | UN | -1 | 28 | 504 | 2000 | 2017 |
| 23 | 8 | 8.4 | Domestic material consumption, Crops (tonnes) | UN | -1 | 28 | 504 | 2000 | 2017 |
| 24 | 8 | 8.4 | Domestic material consumption, Grazed biomass and fodder (tonnes) | UN | -1 | 28 | 504 | 2000 | 2017 |
| 25 | 8 | 8.4 | Domestic material consumption, Wild Catch Harvest (tonnes) | UN | -1 | 28 | 503 | 2000 | 2017 |
| 26 | 8 | 8.5 | Employment rate | Eurostat | 1 | 27 | 486 | 2000 | 2017 |
| 27 | 8 | 8.5 | Long-term unemployment rate | Eurostat | -1 | 28 | 504 | 2000 | 2017 |
| 28 | 9 | 9.2 | Manufacturing value added as a proportion of GDP (%) | UN | 1 | 28 | 504 | 2000 | 2017 |
| 29 | 9 | 9.2 | Manufacturing employment as a proportion of total employment (%) | UN | 1 | 28 | 503 | 2000 | 2017 |
| 30 | 9 | 9.3 | Proportion of small-scale industries in total industry value added (%) | UN | 1 | 28 | 405 | 2000 | 2015 |
| 31 | 9 | 9.4 | Carbon dioxide emissions per unit of GDP (kilogrammes of CO2 per constant 2010 USD) | UN | -1 | 28 | 448 | 2000 | 2015 |
| 32 | 9 | 9.4 | Carbon dioxide emissions per unit of manufacturing value added (kilogrammes of CO2 per constant 2010 United States dollars) | UN | -1 | 28 | 448 | 2000 | 2015 |
| 33 | 9 | 9.5 | Gross domestic expenditure on R&D | Eurostat | 1 | 28 | 504 | 2000 | 2017 |
| 34 | 9 | 9.b | Employment in high- and medium-high technology manufacturing sectors and knowledge-intensive service sectors | Eurostat | 1 | 28 | 280 | 2008 | 2017 |
| 35 | 9 | 9.5 | R&D personnel by sector | Eurostat | 1 | 28 | 504 | 2000 | 2017 |
| 36 | 9 | 9.5 | Patent applications to the European Patent Office (source: EPO) | Eurostat | 1 | 28 | 504 | 2000 | 2017 |
| 37 | 11 | 11.6 | Recycling rate of municipal waste | Eurostat | 1 | 28 | 504 | 2000 | 2017 |
| 38 | 12 | 12.2 | Domestic material consumption, Wood (tonnes) | UN | -1 | 28 | 491 | 2000 | 2017 |
| 39 | 12 | 12.2 | Domestic material consumption, Crop residues (tonnes) | UN | -1 | 28 | 504 | 2000 | 2017 |
| 40 | 12 | 12.2 | Domestic material consumption, Crops (tonnes) | UN | -1 | 28 | 504 | 2000 | 2017 |
| 41 | 12 | 12.2 | Domestic material consumption, Grazed biomass and fodder (tonnes) | UN | -1 | 28 | 504 | 2000 | 2017 |
| 42 | 12 | 12.2 | Domestic material consumption, Wild Catch Harvest (tonnes) | UN | -1 | 28 | 503 | 2000 | 2017 |
| 43 | 12 | 12.5 | Circular material use rate | Eurostat | 1 | 28 | 364 | 2004 | 2016 |
| 44 | 12 | 12.3 | Generation of waste excluding major mineral wastes by hazardousness | Eurostat | -1 | 28 | 196 | 2004 | 2016 |
| 45 | 13 | 13.2 | Greenhouse gas emissions (source: EEA) | Eurostat | -1 | 28 | 756 | 1990 | 2016 |
| 46 | 14 | 14.2 | Surface of marine sites designated under NATURA 2000 (source: DG ENV, EEA) | Eurostat | 1 | 28 | 196 | 2011 | 2017 |
| 47 | 15 | 15.1 | Forest area (thousands of hectares) | UN | 1 | 28 | 112 | 2000 | 2015 |
| 48 | 15 | 15.1 | Average proportion of Freshwater Key Biodiversity Areas (KBAs) covered by protected areas (%) | UN | 1 | 26 | 494 | 2000 | 2018 |
| 49 | 15 | 15.1 | Average proportion of Terrestrial Key Biodiversity Areas (KBAs) covered by protected areas (%) | UN | 1 | 28 | 532 | 2000 | 2018 |
| 50 | 15 | 15.2 | Above-ground biomass in forest per hectare (tonnes per hectare) | UN | 1 | 26 | 104 | 2000 | 2015 |
| 51 | 15 | 15.2 | Forest area certified under an independently verified certification scheme (thousands of hectares) | UN | 1 | 28 | 504 | 2000 | 2017 |
| 52 | 15 | 15.4 | Average proportion of Mountain Key Biodiversity Areas (KBAs) covered by protected areas (%) | UN | 1 | 21 | 399 | 2000 | 2018 |
| 53 | 15 | 15.5 | Red List Index | UN | -1 | 28 | 728 | 1993 | 2018 |
| 54 | 15 | 15.5 | Surface of terrestrial sites designated under NATURA 2000 (source: DG ENV, EEA) | Eurostat | 1 | 28 | 196 | 2011 | 2017 |

**Table SM4- Proportion of synergies, trade-offs and non-classified correlations by SDG pairs**

| SDG pair | | | | | Synergies (green) | Trade-offs (orange) | Non- classified (yellow) |  | SDG pair | | | | | Synergies (green) | Trade-offs (orange) | Non- classified (yellow) |
| --- | --- | --- | --- | --- | --- | --- | --- | --- | --- | --- | --- | --- | --- | --- | --- | --- |
| SDG | 2 | x | SDG | 2 | 47% | 42% | 10% |  | SDG | 11 | x | SDG | 12 | 45% | 31% | 24% |
| SDG | 2 | x | SDG | 8 | 42% | 33% | 26% |  | SDG | 11 | x | SDG | 15 | 79% | 9% | 12% |
| SDG | 2 | x | SDG | 9 | 50% | 38% | 11% |  | SDG | 12 | x | SDG | 2 | 44% | 35% | 21% |
| SDG | 4 | x | SDG | 2 | 63% | 28% | 10% |  | SDG | 12 | x | SDG | 4 | 45% | 34% | 22% |
| SDG | 4 | x | SDG | 4 | 63% | 23% | 15% |  | SDG | 12 | x | SDG | 7 | 50% | 32% | 17% |
| SDG | 4 | x | SDG | 9 | 63% | 28% | 9% |  | SDG | 12 | x | SDG | 12 | 50% | 28% | 23% |
| SDG | 6 | x | SDG | 2 | 59% | 24% | 17% |  | SDG | 13 | x | SDG | 2 | 55% | 21% | 24% |
| SDG | 6 | x | SDG | 4 | 69% | 26% | 4% |  | SDG | 13 | x | SDG | 4 | 60% | 35% | 5% |
| SDG | 6 | x | SDG | 6 | 72% | 7% | 21% |  | SDG | 13 | x | SDG | 6 | 71% | 8% | 21% |
| SDG | 6 | x | SDG | 12 | 38% | 42% | 21% |  | SDG | 13 | x | SDG | 7 | 94% | 0% | 6% |
| SDG | 7 | x | SDG | 2 | 59% | 29% | 12% |  | SDG | 13 | x | SDG | 8 | 38% | 33% | 30% |
| SDG | 7 | x | SDG | 4 | 72% | 23% | 5% |  | SDG | 13 | x | SDG | 9 | 63% | 22% | 15% |
| SDG | 7 | x | SDG | 6 | 77% | 12% | 10% |  | SDG | 13 | x | SDG | 11 | 82% | 0% | 18% |
| SDG | 7 | x | SDG | 7 | 94% | 3% | 4% |  | SDG | 13 | x | SDG | 12 | 57% | 16% | 28% |
| SDG | 7 | x | SDG | 8 | 43% | 36% | 20% |  | SDG | 13 | x | SDG | 15 | 66% | 10% | 24% |
| SDG | 7 | x | SDG | 9 | 66% | 25% | 9% |  | SDG | 14 | x | SDG | 2 | 59% | 41% | 0% |
| SDG | 7 | x | SDG | 15 | 74% | 16% | 10% |  | SDG | 14 | x | SDG | 4 | 67% | 33% | 0% |
| SDG | 8 | x | SDG | 4 | 54% | 22% | 24% |  | SDG | 14 | x | SDG | 6 | 50% | 50% | 0% |
| SDG | 8 | x | SDG | 6 | 35% | 39% | 26% |  | SDG | 14 | x | SDG | 7 | 84% | 16% | 0% |
| SDG | 8 | x | SDG | 8 | 36% | 32% | 32% |  | SDG | 14 | x | SDG | 8 | 60% | 40% | 0% |
| SDG | 8 | x | SDG | 12 | 68% | 17% | 14% |  | SDG | 14 | x | SDG | 9 | 59% | 41% | 0% |
| SDG | 9 | x | SDG | 6 | 55% | 28% | 17% |  | SDG | 14 | x | SDG | 11 | 75% | 25% | 0% |
| SDG | 9 | x | SDG | 8 | 40% | 34% | 26% |  | SDG | 14 | x | SDG | 12 | 48% | 52% | 0% |
| SDG | 9 | x | SDG | 9 | 52% | 36% | 13% |  | SDG | 14 | x | SDG | 13 | 100% | 0% | 0% |
| SDG | 9 | x | SDG | 12 | 42% | 37% | 21% |  | SDG | 15 | x | SDG | 2 | 59% | 28% | 13% |
| SDG | 9 | x | SDG | 15 | 62% | 27% | 12% |  | SDG | 15 | x | SDG | 4 | 72% | 15% | 13% |
| SDG | 11 | x | SDG | 2 | 67% | 23% | 10% |  | SDG | 15 | x | SDG | 6 | 71% | 12% | 17% |
| SDG | 11 | x | SDG | 4 | 79% | 9% | 12% |  | SDG | 15 | x | SDG | 8 | 44% | 29% | 27% |
| SDG | 11 | x | SDG | 6 | 79% | 14% | 7% |  | SDG | 15 | x | SDG | 12 | 48% | 31% | 21% |
| SDG | 11 | x | SDG | 7 | 91% | 1% | 8% |  | SDG | 15 | x | SDG | 14 | 70% | 30% | 0% |
| SDG | 11 | x | SDG | 8 | 44% | 29% | 28% |  | SDG | 15 | x | SDG | 15 | 76% | 16% | 8% |
